# Comparative genomics of space-resilient *Chroococcidiopsis* cyanobacteria reveals a core genetic repertoire supporting extreme tolerance towards desert and non-Earth conditions

**DOI:** 10.1093/femsmc/xtag036

**Published:** 2026-06-13

**Authors:** Gabriele Rigano, Daniela Billi

**Affiliations:** Department of Biology, University of Rome “Tor Vergata”, Via della ricerca scientifica 0, 00133 Rome, Italy; Department of Physics, PhD programme in Space Science and Technology, University of Trento, Via Sommarive 14, 38123 Trento, Italy; Department of Biology, University of Rome “Tor Vergata”, Via della ricerca scientifica 0, 00133 Rome, Italy

**Keywords:** desert cyanobacteria, desiccation, DNA repair, secondary metabolism, space exposure, extreme environments

## Abstract

Desert cyanobacteria of the *Chroococcidiopsis* genus, due to their remarkable desiccation and radiation tolerance, are considered model candidates in the field of astrobiology. For this reason, three strains, namely *Chroococcidiopsis* sp. CCMEE 029, 057, and 064 have been exposed in the dried state to space- and Mars-like conditions throughout laboratory simulations and real space exposure using the EXPOSE facility installed outside the International Space Station. However, how they can recover upon rehydration and repair the damage accumulated under extreme conditions on Earth and in space remains to be fully elucidated through omics-based investigations. Hence, comparative genomics of these three *Chroococcidiopsis* strains offered a powerful lens to explore the genetic components of their capability to persist under extreme conditions. The analysis of newly obtained gapless genome assemblies of the laboratory-maintained reference strains of CCMEE 029, 057, and 064 allowed the identification of conserved and non-shared genes involved in reactive oxygen species detoxification, desiccation tolerance, DNA protection, and repair. Moreover, a biosynthetic gene cluster for scytonemin production was identified in every strain, while strain CCMEE 057 also harbored the genes for the biosynthesis of a mycosporine-like amino-acid. Such insights are crucial for understanding the adaptation strategies employed by microorganisms to survive in dry, radiation-intense environments, offering clues about the potential for life beyond Earth.

## Teaser

By comparing the genomes of three highly space-resilient desert cyanobacteria, this research reveals the biological tools that allow them to survive severe drying, intense radiation, and extreme space conditions.

## Introduction

Cyanobacteria from the *Chroococcidiopsis* genus are among the most radiation-tolerant photosynthetic microorganisms (Billi et al. 2000, Cox and Battista 2005) inhabiting some of the harshest environments on Earth, such as hyper-arid hot deserts and ice-free regions, where they colonize lithic niches and spend most of the time in a dried, ametabolic state (Imre Friedmann and Ocampo-Friedmann 1995). Their remarkable ability to persist under long-term water deprivation, oxidative stress, and high levels of ultraviolet and ionizing radiation has positioned *Chroococcidiopsis* as a key model system for studying microbial survival strategies under multiple environmental stressors, thus providing an experimental model for astrobiology studies dealing both with the limits of life as we know it and the search for it outside of our planet (Baqué et al. 2013, Billi et al. 2017, Billi 2020).

Desiccation represents one of the most severe stress conditions affecting cellular integrity, as it leads to extensive oxidative damage, protein denaturation, membrane destabilization, and DNA lesions (Billi and Potts 2002). In cyanobacteria, desiccation tolerance is supported by a multifaceted network of physiological and molecular adaptations, including the accumulation of compatible solutes, efficient DNA protection and repair systems, robust antioxidant defenses, and specialized photoprotective mechanisms (Singh 2018, Rajaram et al. 2020, Rai et al. 2025). Previous studies have highlighted the central role of trehalose, sucrose, and other osmoprotectants, together with antioxidant enzymes, molecular chaperones, and UV-screening compounds like scytonemins and mycosporine-like amino acids (MAAs) in enabling *Chroococcidiopsis* strains to withstand extreme dehydration and irradiation (Boukar et al. 2024, Di Stefano et al. 2025).

Comparative genomics offers a powerful framework to investigate both conserved and strain-specific features that shape stress tolerance and ecological adaptation (Cassier-Chauvat et al. 2016, Chen et al. 2021). While core genome analyses can reveal fundamental biological processes shared across lineages, the accessory and singleton gene pools often encode functions associated with environmental specialization, metabolic versatility and adaptive strategies. In cyanobacteria, comparative analyses have proven particularly informative in uncovering differences in antioxidant capacity, DNA repair repertoires, secondary metabolite biosynthesis and regulatory networks, thereby providing insights into how closely related strains diversify in response to distinct ecological pressures (Cassier-Chauvat et al. 2016, Di Stefano et al. 2025).

In particular, *Chroococcidiopsis* strains CCMEE 029, 057, and 064, selected for the different modalities of rock colonization (cryptoendolithic, chasmoendolithic, and hypolithic respectively) and different types of colonized rocks (granite or sandstone), have been extensively used in experimental astrobiology because of their desiccation and radiation tolerance (Billi et al. 2017, Billi 2026). These strains have demonstrated remarkable resilience in laboratory and space experiments, supporting their relevance as model organisms to define the boundaries of habitability of other worlds and to investigate the preservation of potential biosignatures (Fagliarone et al. 2020). These strains were exposed to space and Mars-like simulations as dried biofilms during the BOSS (Biofilm Organisms Surfing Space) space mission performed onboard the EXPOSE-R2 facility installed outside the International Space Station (Cottin and Rettberg 2019). Post-flight analyses revealed survivors in the bottom layers of the biofilms that were shielded against UV radiation by top-layer cells (Billi et al. 2019a). In addition, *Chroococcidiopsis* sp. CCMEE 029 was exposed to space and Mars-like simulations during the BIOMEX experiment onboard the EXPOSE-R2 facility (de Vera et al. 2019). Post-flight analyses identified survivors that were shielded against UV radiation by the mixing with Mars regolith simulants (Billi et al. 2019b). How desert strains of *Chroococcidiopsis* exposed to non-Earth conditions in the air-dried state are able to recover upon rehydration and repair the accumulated damage remains to be fully elucidated through omics-based investigations.

In this study, a comparative genome analysis of the three experimentally tested space-resistant *Chroococcidiopsis* sp. CCMEE 029, 057, and 064 was performed by integrating gene content analysis, functional annotation, and metabolic pathway reconstruction to investigate the core and accessory proteomes associated with desiccation resistance, DNA damage protection and repair, and oxidative stress mitigation. In addition, we performed targeted genome mining to identify and compare biosynthetic gene clusters (BGCs) involved in the production of UV-protective secondary metabolites, including scytonemin and MAAs.

By elucidating both conserved and strain-specific genomic traits, this work provides new insights into the molecular strategies that underpin resilience in the dried state to space- and Mars-like conditions in *Chroococcidiopsis* and expands our current understanding on how their genome plasticity supports the colonization of some of the most inhospitable environments on Earth.

## Materials and methods

### Strain cultivation, DNA extraction, and sequencing

*Chroococcidiopsis* sp. CCMEE 057 and CCMEE 064 were collected in 1969 by E. Imre Friedmann from chasmoendolithic and hypolithic communities in the Sinai Desert respectively and isolated by Roseli Ocampo-Friedmann, while *Chroococcidiopsis* sp. CCMEE 029 was collected in 1969 by E. Imre Friedmann from cryptoendolithic communities in the Negev desert and then isolated by Roseli Ocampo-Friedmann. Starting from 2006 these strains are maintained through sequential culturing at the University of Rome Tor Vergata as part of the Culture Collection of Microorganisms from Extreme Environments (CCMEE) established by E. Imre Friedmann. Cyanobacterial cultures of *Chroococcidiopsis* sp. CCMEE 057 and CCMEE 064 were incubated for 21 days in BG-11 medium, at 25°C under a constant photon flux density of 10 μmol/m^2^/s, which was provided by a white LED light (4000 K, OSRAM, MI, Italy).

Genomic DNA was sequenced through the Oxford Nanopore and Illumina MiSeq technologies. For Oxford Nanopore libraries, samples were labelled with a rapid barcoding kit SQK-RBK004 to then be sequenced through the LSK-SQK109 ligation kit. Base calls were performed with guppy v4.4.1.

Illumina libraries were prepared through the Kapa Hyperplus library kit (Roche Molecular Systems Inc., Pleasanton, CA, USA) following the manufacturer’s instructions. The final pooled library was quantified with qPCR and sequenced employing the MiSeq Illumina platform using the chemical V3 PE 2 × 300. A library size of 400 bp was then detected through the Agilent 2100 TapeStation.

Sequencing libraries were submitted to the SRA database with the following accessions:

SRR36983983-85, SRR37017621, SRR36989150-51.

*Chroococcidiopsis* sp. CCMEE 029 previously sequenced Illumina and Nanopore libraries were downloaded from the SRA database (SRA study accession: SRP328671).

Genome assemblies and annotations from this study can be found at the dedicated GitHub repository https://github.com/GabrieleRigano99/Comparative_genomics_Chroococcidiopsis_029_057_064.

### Bioinformatic analysis

Raw reads' quality check was performed using FastQC version 0.11.8 (Andrews 2010) and longQC v1.2.1 (Fukasawa et al. 2020) for Illumina short reads and Nanopore long reads respectively.

Illumina reads were trimmed with fastp v0.20.4 (Chen et al. 2018) with “–detect_adapter_for_pe” parameter.

Draft genome assemblies were generated with Flye v2.9.2-b1786 (Kolmogorov et al. 2020) with “–meta –scaffold” parameters using Nanopore reads; subsequently trimmed Illumina reads were aligned to draft genome assemblies with the bwa—samtools view—samtools sort (Li 2013, Danecek et al. 2021) workflow and finally polished the draft genomes with 5 iterations of Pilon v1.24 (Walker et al. 2014). Genome assembly contiguity and contaminants removal was assessed through manual inspection with Bandage v0.9 (Wick et al. 2015).

Assembly quality was evaluated with compleasm v0.2.7 (Huang and Li 2023) on the cyanobacteriota_odb12 level and through quast v5.3.0 (Gurevich et al. 2013)

Polished genome assemblies were then annotated with PROKKA v1.14.6 for structural annotation; EggNOG mapper v2.1.12 and the EggNOG 5.0.2 database, InterProScan v.5.74–105.0, SignalP6.0, Phobius v1.0.1 BlastKoala, and the CAZyme database were employed for functional annotation (Käll et al. 2004, Jones et al. 2014, Huerta-Cepas et al. 2019, Cantalapiedra et al. 2021, Drula et al. 2022, Teufel et al. 2022); the AGAT toolkit was employed to implement all functional annotations into the final GFF files (Dainat et al. 2026).

In order to explore and compare the genomic landscape of the investigated *Chroococcidiopsis* strains, we employed a pangenome-based strategy through the RIBAP v1.1.1 and OrthoVenn3.0 software with default parameters (Sun et al. 2023, Lamkiewicz et al. 2024). Moreover, we employed the JolyTree v.1.1b software (Criscuolo 2019) with parameters “-r 1000” to create a phylogenetic tree based on all the genome assemblies of the *Chroococcidiopsis* genus available in the NCBI database (Downloaded 21/04/2026). An average nucleotide identity (ANI) matrix was produced with the fastANI v.1.34 tool (Jain et al. 2018) among the three investigated strains to infer genetic diversity.

Subsequently, through manual inspection, EggNOG5 orthology database and literature review, we investigated the genomic components related to desiccation, DNA damage and repair, antioxidants and photoprotective pigments production such as scytonemin and MAAs.

Scytonemin and MAAs biosynthetic gene clusters were manually searched using as reference the filamentous cyanobacterium *Nostoc punctiforme* PCC 73102 (Refseq accession: GCF_000020025.1).

BGCs comparative analysis and visualization was executed through the GeneViewer v0.11.1 software (van der Velden 2025).

## Results

### Gene content and metabolic profiling of *Chroococcidiopsis* sp. CCMEE 029, 057, and 064 strains

Total mean reads coverage for *Chroococcidiopsis* sp. CCMEE 029, 057, and 064 was 54X, 55X, and 54X, respectively (24X, 11X, and 22X Illumina libraries, respectively).

A single circular chromosome-level assembly was obtained for each strain, observing comparable genome sizes: 5.72, 5.20, and 5.08 Mbp for *Chroococcidiopsis* sp. CCMEE 029, 057, and 064, respectively, with a GC % of 46.17, 46.44, and 46.23.

Genome assembly quality evaluated with compleasm resulted in more than 99% completeness in the category “complete and single copy” for every genome.

**Figure 1:**
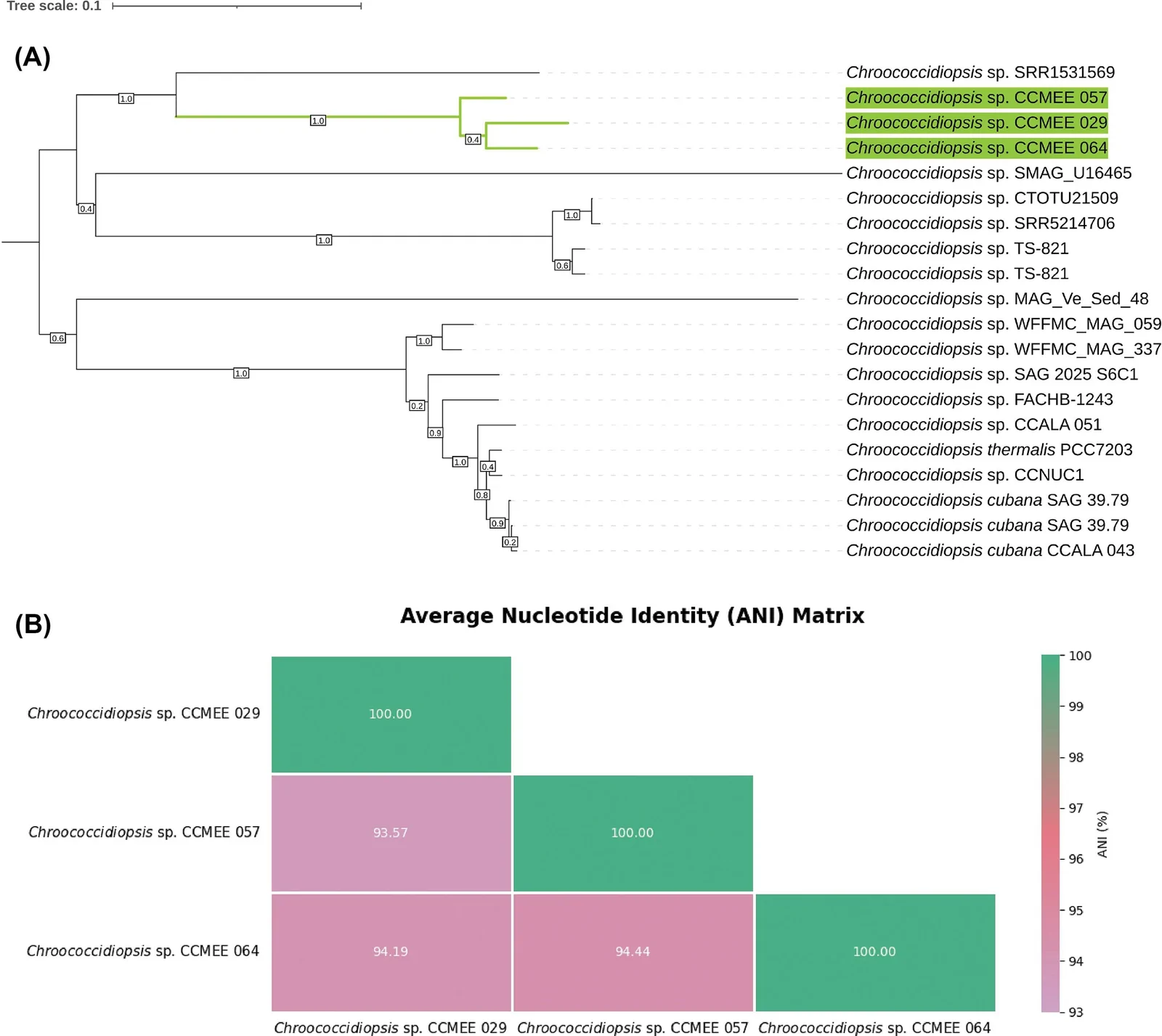
Whole genome-based phylogenetic tree of all *Chroococcidiopsis* species sequenced to date separating *Chroococcidiopsis* sp. CCMEE 029, 057, and 064 into a well defined cluster (A); ANI distance across *Chroococcidiopsis* sp. CCMEE 029, 057, and 064 (B).

The phylogenetic analysis based on all available genome assemblies to date placed *Chroococcidiopsis* sp. CCMEE 029, 057, and 064 into a well separated clade, distant from the model organism *Chroococcidiopsis thermalis* PCC7203, while the ANI divergence between the investigated strains resulted in a minimum of 93.57 to a maximum of 94.44, indicating a slight level of genetic diversity (Fig. 1).

From a gene content point of view, we found a total of 5.615, 5.122, and 4.812 genes (CCMEE 029, 057, 064, respectively), of which 5.564, 5.071, and 4.759 were protein-coding, while 44 tRNAs were predicted in every strain and a total of 7, 7, and 8 rRNAs were also identified.

**Figure 2:**
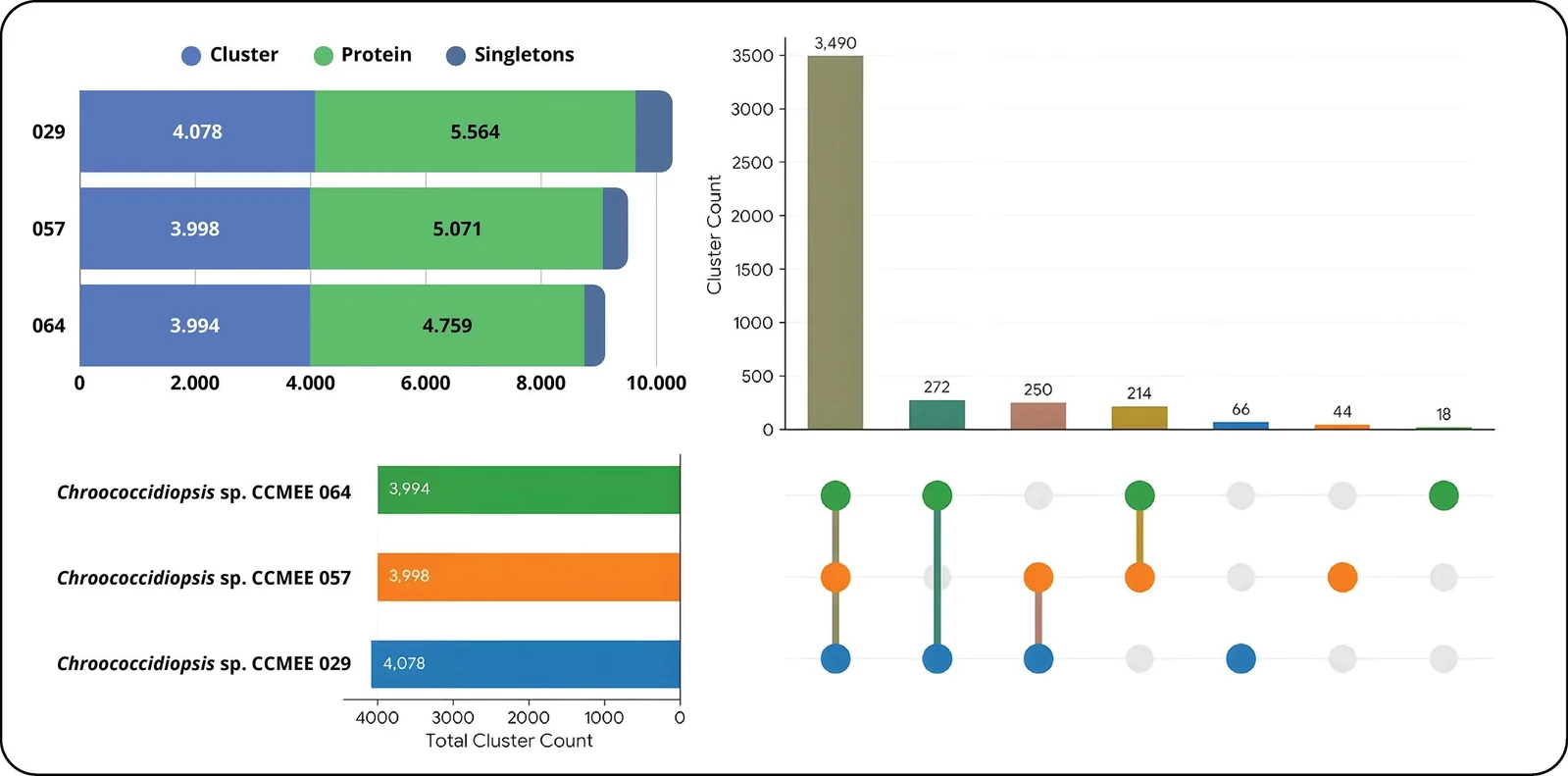
Upset plot of core, accessory, and singleton protein clusters among *Chroococcidiopsis* sp. CCMEE 029, 057, and 064.

The comparative analysis on protein-coding genes revealed a high number of shared genes, making up a core proteome of 3.490 protein clusters, amounting to 80.2% of the investigated proteomes, while 736 protein clusters (16.9%) consisted in the accessory proteome and 128 proteins accounted for 2.9% of all singleton proteins (Fig. 2).

**Figure 3:**
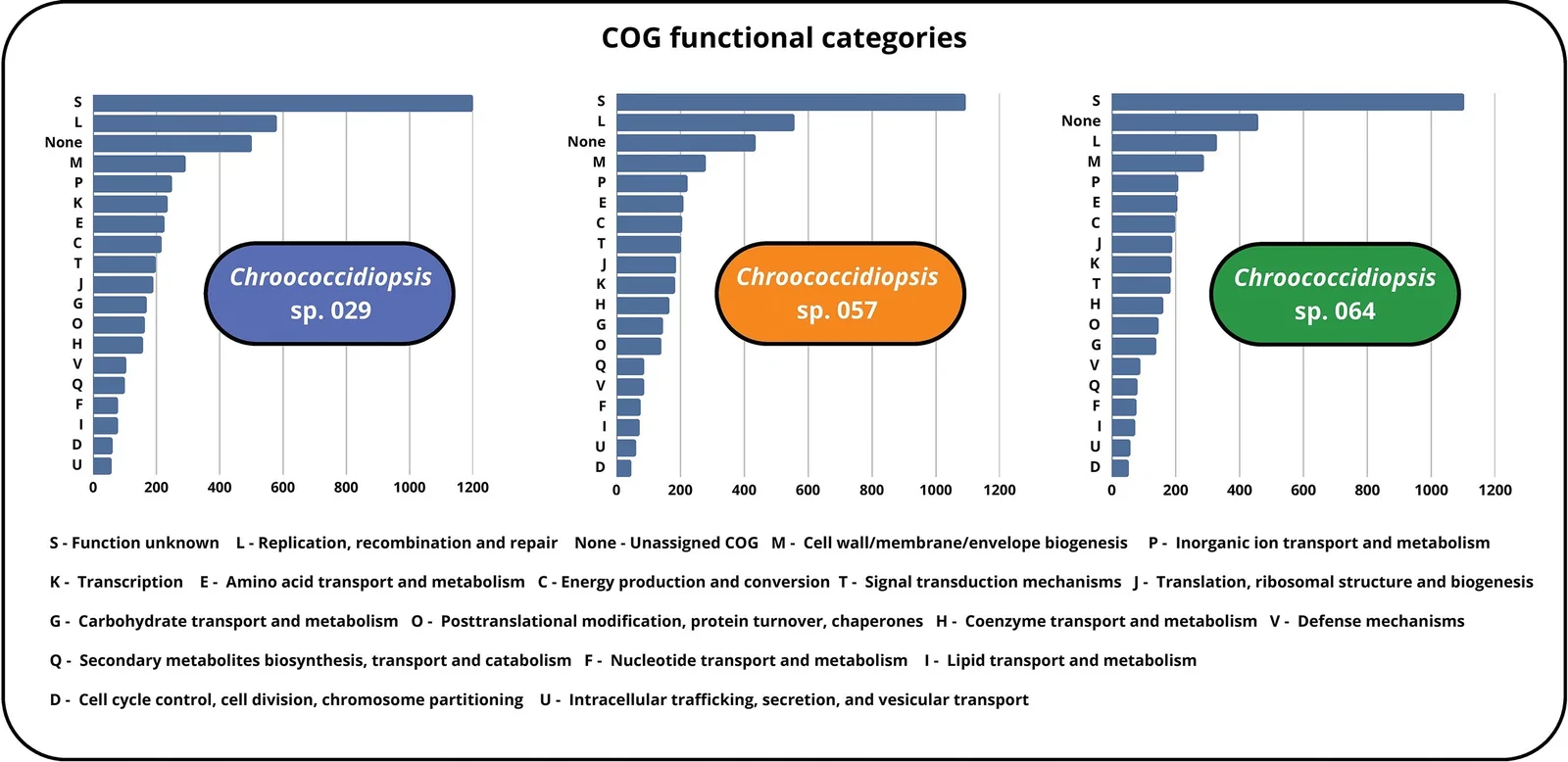
COG functional categories profiles of *Chroococcidiopsis* sp. CCMEE 029, 057, and 064 genomes.

Regarding the functional aspect of the investigated genomes, the EggNOG software assigned as the most abundant annotated cluster of Orthologous Groups (COG) term the replication, recombination and repair (L) category, with 580, 557, and 329 proteins for 029, 057, and 064 respectively. A similar trend was observed in cell wall/membrane/envelope biogenesis (M), inorganic ion transport and metabolism (P), amino acid transport and metabolism (E), energy production and conversion (C), and signal transduction mechanisms (T) (Fig. 3).

In addition, Kyoto Encyclopedia of Genes and Genomes (KEGG) functional categories annotation highlighted the shared functional aspect across the *Chroococcidiopsis* genomes regarding energy metabolism, carbohydrate and glycan biosynthesis, amino acid, cofactors, and vitamins metabolism as well as environmental information processing.

**Figure 4:**
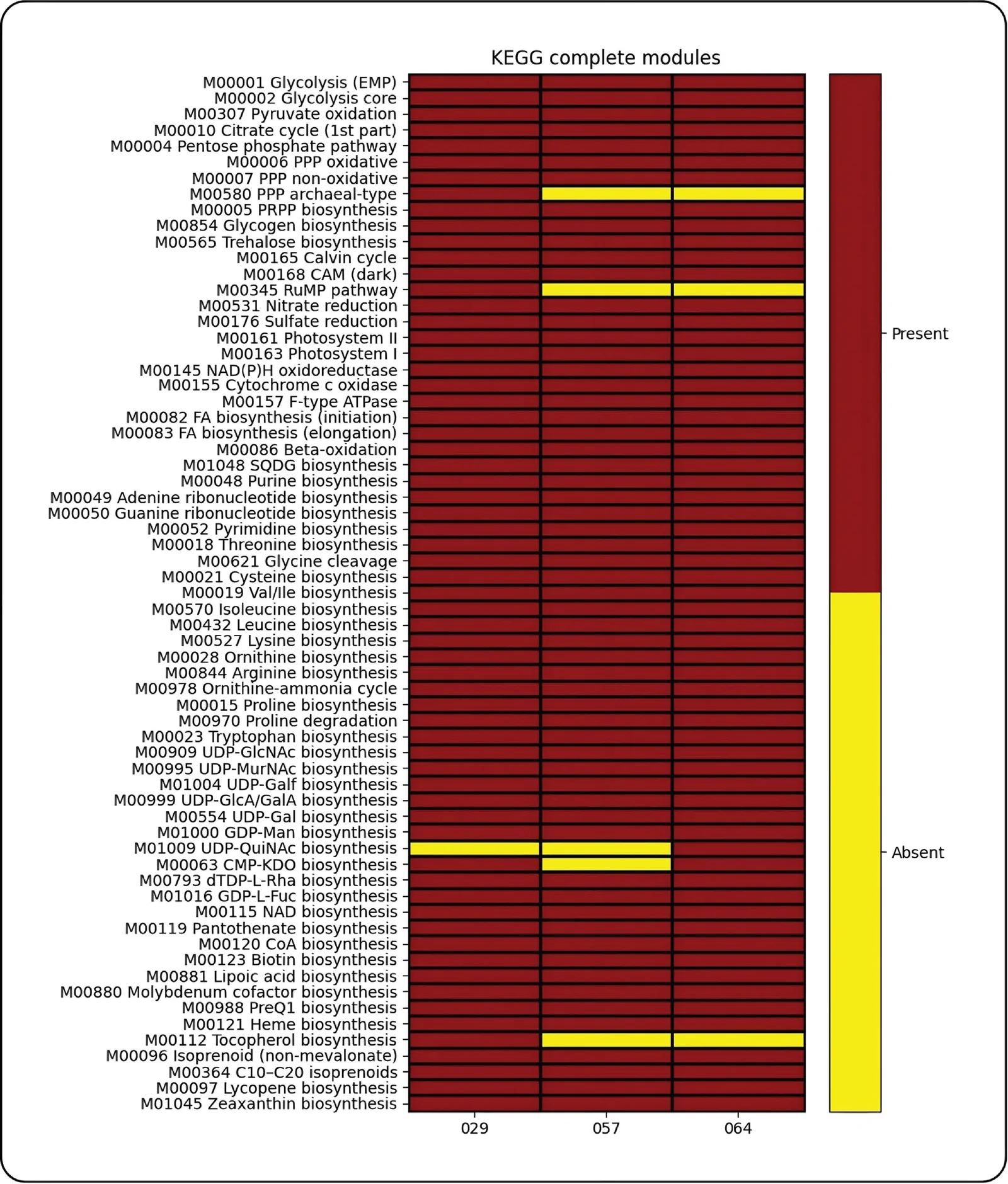
Comparative analysis of metabolic pathways completeness across *Chroococcidiopsis* sp. CCMEE 029, 057, and 064 strains.

Furthermore, from the KEGG pathway reconstruction analysis, a total of 60 out of 65 complete modules were shared (Fig. 4), including carbohydrate metabolism pathways such as trehalose and glycogen biosynthesis or carotenoids like zeaxanthin and lycopene biosynthesis, while only *Chroococcidiopsis* sp. CCMEE 029 was found to harbour the genes (*hxlA, hxlB*) for the ribulose monophosphate pathway (RuMP) and to be able to produce tocopherol (vitamin E). On the other hand, *Chroococcidiopsis* sp. CCMEE 064 was predicted to be the only strain capable of synthesizing the UDP-QuiNAc nucleotide sugar, as its genome contained the *wbpM* and *wbpV* genes.

Finally, *Chroococcidiopsis* sp. CCMEE 057 was the only one predicted to be unable to produce cytidine monophosphate-3-deoxy-D-manno-octulosonic acid (CMP-KDO), as it lacks the *kdsD* gene encoding for arabinose 5-phosphate isomerase, the first enzyme of the M00063 module required to transform D-Ribulose-5P into D-Arabinose-5P.

### Genetic repertoire associated with desiccation resistance

The genetic repertoire relative to desiccation showed a solid core represented by genes for trehalose biosynthesis, namely *treS, treZ*, and *treY* (with *Chroococcidiopsis* sp. CCMEE 057 harboring three copies of *treS*), sucrose biosynthesis with *susA* present in two copies in distal parts of their genomes and the *mfppA* gene, (which is phylogenetically close to *sps* and *spp* genes) present in every genome, acting as a dual-functioning sucrose synthase and phosphatase, according to bioinformatic predictions (Torres and Salerno 2007, Klähn and Hagemann 2011).

Additionally, we identified the required genes for proline production (*proA, proB*, and *proC*), stress (Klähn and Hagemann 2011), which is also a secondary compatible solute synthesized in response to osmotic and drought stress. No genes related to glucosylglycerol and glycine betaine production were found from our analysis, as *ggpP, ggpS, gsmT*, and *sdmT* genes were not identified. A complete analysis with KEGG, EggNOG IDs, and EC_numbers of all investigated genes is found in Supplementary Table S1.

### DNA damage-protection, repair-related genes, and dNTP pools maintenance genes

Among DNA protection-associated genes, damage protection during starvation encoding genes (*dps*) were found in multiple copies, which according to the *Nostoc punctiforme* PCC 73102 nomenclature were identified as orthologous to NpDps1 (*Npun_R3258*), NpDps2 (*Npun_F3730*), NpDps4 (*Npun_R5799*), and NpDps5 proteins (*Npun_F6212*) (Ekman et al. 2014), while a different *dps* orthologous to *Gloeobacter violaceus* and *Chroococcidiopsis thermalis* was also identified (*glr4292* and *Chro_1299*, respectively; EggNOG5 orthology ID: ENOG501G4NT).

**Figure 5:**
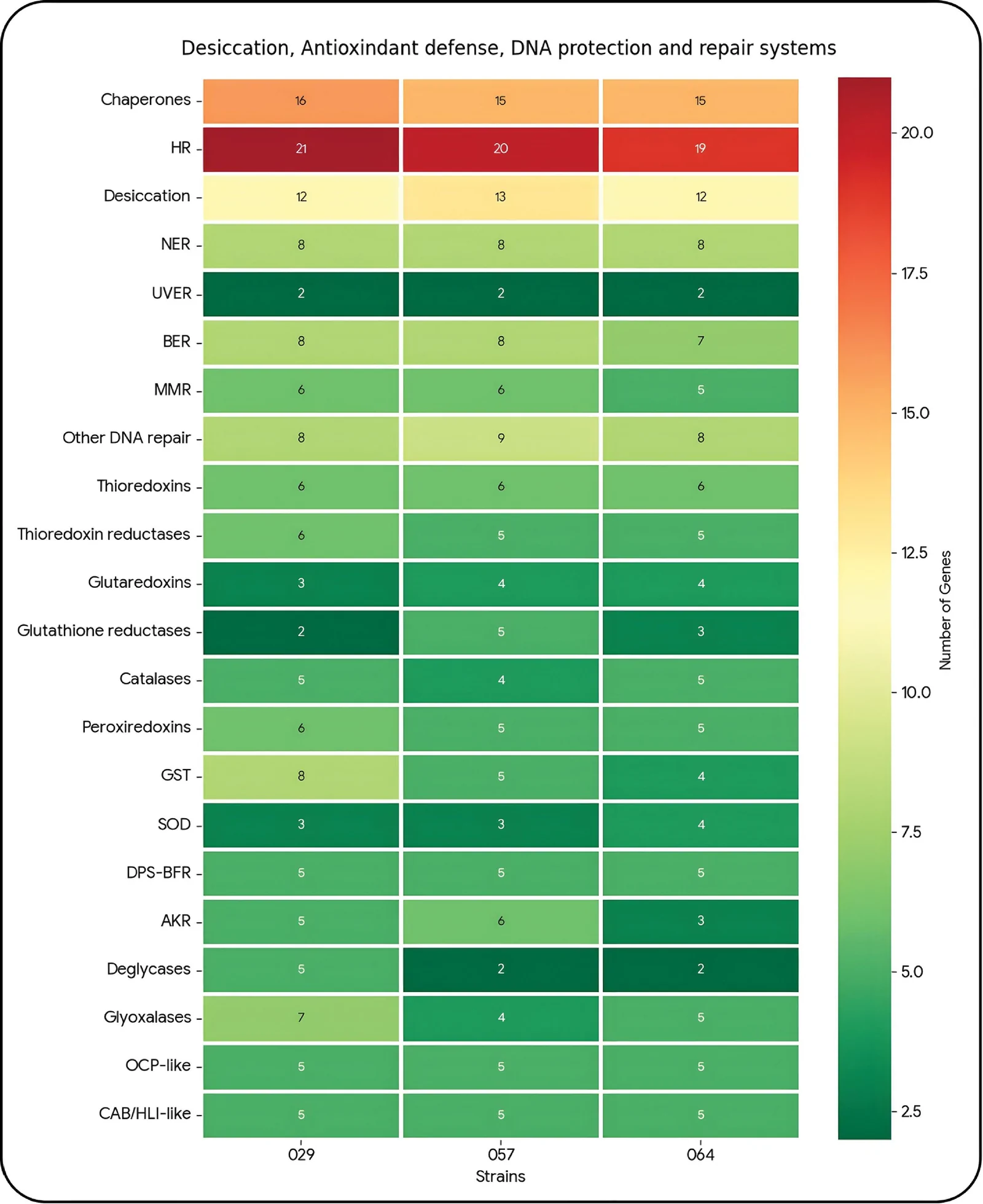
Comparative analysis of desiccation, antioxidant defense, DNA protection, and repair genes found across the investigated *Chroococcidiopsis* strains.

Regarding genes related to DNA repair mechanisms, we identified a consistent repertoire of genes belonging to the Nucleotide Excision Repair (NER), Base Excision Repair (BER), mismatch repair (MMR), Double Strand Break (DBS), the UVDE-mediated excision repair (Goosen and Moolenaar 2008), together with other genes encoding DNA repair proteins like *radC* and nucleoside kinases (*adk, gmk, tmk, ndk*, etc.; Fig. 5).

### Enzymatic and non-enzymatic antioxidants landscape

A considerable repertoire of antioxidant mechanisms was identified of both enzymatic and non-enzymatic nature.

In particular, manganese and copper-zinc binding superoxide dismutases (*sodA2.1, sodA2.2*, and *sodC* in 1 copy) were found in every genome, with *Chroococcidiopsis* sp. CCMEE 064 being the only strain to contain also the *sodB* gene, encoding for the iron binding SOD. Manganese catalases (*ydbD/MnCat*) occurred in two copies, like monofunctional *katE* catalases, while *Chroococcidiopsis* sp. CCMEE 057 was the only devoid of the *katG* catalase-peroxidase encoding gene.

Regarding peroxiredoxins, five types were identified, among which the hybrid peroxiredoxin hyPrx5 (*pgdx*), thioredoxin-dependent peroxiredoxin *prxQ*, peroxiredoxin *osmC*, and 1- and 2-cys peroxiredoxins (*prx* and *prxU*), where *Chroococcidiopsis* sp. 029 harbored 2 copies of *prx*.

Furthermore, the glutaredoxin gene content was made up of both monothiol (*grxC*) and dithiol glutaredoxins (*grxA, grxB*), classified according to the CXXC domain they contained, where the CGFS motif represents *grxC*, CSYC and CPFC represent *grxA* and *grxB* respectively (Pérez-Pérez et al. 2009). In this case, *Chroococcidiopsis* sp. CCMEE 029 did not contain any *grxA* gene.

Thioredoxins were also found in every investigated genome in the same ratio, including *trxA, trxB, trxC*, and *trxQ*. Similarly to thioredoxin-reductases in which *Chroococcidiopsis* sp. CCMEE 029 lacked one annotated as a diflavin-linked disulfide oxidoreductase (Mallén-Ponce et al. 2022).

The glutathione-related gene pool, comprising glutathione reductases (GR) and glutathione S transferases (*gst*) was the most divergent group among the enzymatic antioxidants category, as *Chroococcidiopsis* sp. CCMEE 057 contained five copies of the *garB/gor* gene for GR, and *Chroococcidiopsis* sp. CCMEE 029 harbored almost double the amount of *gst* genes compared to the other species.

A similar pattern was then observed for aldo-keto reductases (*iolS, yhdN*), deglycases (*yhbO, hchA*), and for genes related to the glyoxalase system, fundamental for methylglyoxal detoxification. In particular, we found a putative glyoxalase 3 (*glyIII*) only in *Chroococcidiopsis* sp. CCMEE 029, together with five copies of *gloA* and two of *gloB*, but surprisingly we did not identify any D-lactate dehydrogenase (*ldh*) in this strain, while it was found in *Chroococcidiopsis* sp. CCMEE 057 and 064. Chaperones like *groEL, groES, hspA, clpB, dnaJ* and *dnaK* were abundant in all the strains under study.

Finally, the non-enzymatic antioxidant gene reservoir was particularly rich in orange carotenoid binding-like proteins involved in non-photochemical quenching, and protection toward reactive oxygen species, especially singlet oxygen (*ocp-like*, five copies). Specifically, we found the presence of HCP1, HCP2, HCP3, HCP4, and CTDH classes (Melnicki et al. 2016, Bao et al. 2017) and high light inducible-like proteins, containing the characteristic conserved domain of chlorophyll binding (IPR022796) (*cab/hli*, four copies), contributing to maintaining the photosynthetic apparatus integrity and chlorophyll-protein biogenesis.

### CAZyme repertoire comparison

The comparative analysis of carbohydrate-active enzymes (CAZymes) revealed a conserved glycosylation and carbohydrate metabolism potential across *Chroococcidiopsis* sp. CCMEE 029, 057, and 064 (Supplementary Table S2). Glycosyltransferase families GT2 and GT4 were the most abundant in all strains, reflecting a shared core capacity for polysaccharide biosynthesis, cell envelope construction, and storage carbohydrate metabolism, including glycogen and compatible solutes such as trehalose. Members of the GH13 family, involved in glycogen and starch remodeling, were present at comparable levels in the three genomes, indicating conserved carbohydrate mobilization strategies during nutrient limitation or stress. Similarly, the abundance of SLH domain-containing proteins was stable across strains, suggesting conserved mechanisms for anchoring proteins to the cell surface. Notable strain-specific differences were observed in GT2_Glyco_transf_2 and GT27 families: *Chroococcidiopsis* sp. CCMEE 064 showed a marked reduction in these enzyme categories, which are associated with protein and cell surface glycosylation. These differences could point to variation in cell envelope glycosylation and surface architecture among closely related strains, potentially reflecting niche-specific adaptation.

### Genome mining of scytonemin and MAAs biosynthetic gene clusters

**Figure 6:**
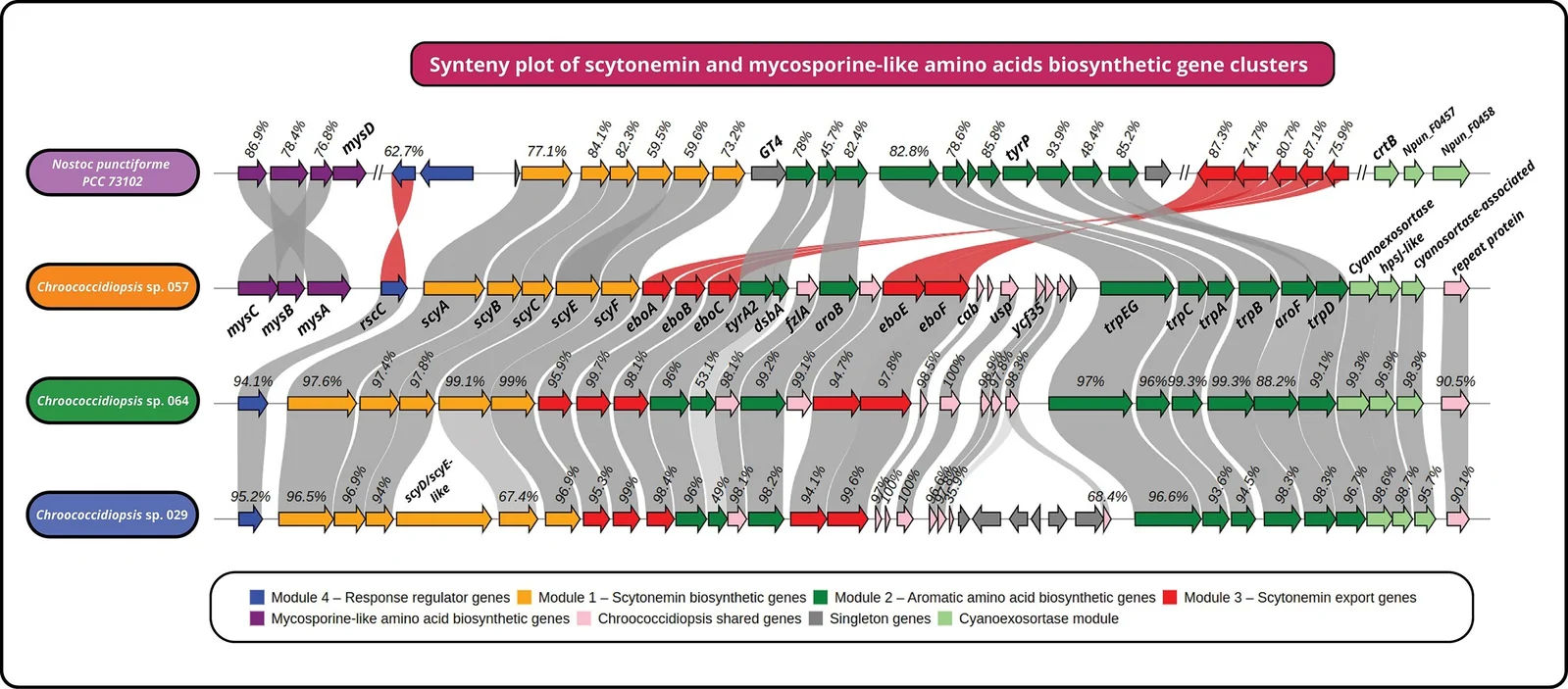
Synteny plot showing scytonemin and MAAs gene clusters with protein similarities referred to *Chroococcidiopsis* sp. CCMEE 057, taken as reference. Module 1 (in yellow) contains the genes for the biosynthesis of scytonemin monomers and dimers; Module 2 (in darkgreen) is responsible for aromatic amino acid substrates biosynthesis; Module 3 (in red) is made up of the genes for the export of scytonemin; Module 4 is responsible for response regulation to UV radiation; Cyanoexosortase module (in lightgreen) responsible for the export of scytonemin; MAAs module (in purple) containing MAAs biosynthetic genes; Shared *Chroococcidiopsis* genes (in pink) characterized by genes present in all investigated strains; Singleton genes module (in grey) containing genes present only in one species.

The genome mining analysis, targeted at scytonemin and MAAs biosynthetic gene clusters discovery, revealed a well-conserved and contiguous region harboring all the genes necessary for scytonemin production in all *Chroococcidiopsis* strains, which also contained some additional genes related to its export annotated as cyanoexosortases, which have been recently identified in *Nostoc punctiforme* PCC 73102 in this regard (Parrett et al. 2025) (Fig. 6).

Interestingly, we found that not only *Chroococcidiopsis* sp. CCMEE 057 possessed the genes for the production of mycosporine-glycine (namely *mysA, mysB*, and *mysC*), but they also directly preceded the scytonemin cluster by ∼950 bps, although co-regulation with scytonemin BGC has yet to be investigated.

## Discussion

In this study we provide a comparative genomic framework to investigate the genetic components that confer remarkable resilience to *Chroococcidiopsis* sp. CCMEE 029, 057, and 064: astrobiologically relevant cyanobacteria that survive in harsh deserts on Earth and the exposure as dried cells to space and Mars-like conditions throughout multiple laboratory simulations and space exposure (Billi 2026). By leveraging chromosome-level assemblies and a pangenome-based approach, we assessed the presence of conserved molecular strategies essential to face desiccation combined with exposure to non-Earth conditions, linked to genetic determinants associated with oxidative stress mitigation, DNA protection and repair, carbohydrate metabolism, and photoprotection. Also, the phylogenetic analysis positioned these strains in a distinct branch across *Chroococcidiopsis* species, pointing to a considerable level of genetic variability in this genus.

### A highly conserved genomic core highlights shared metabolic strategies for stress tolerance

The identification of a large conserved core proteome, accounting for over 80% of the protein-coding genes across the three strains, highlights the existence of a shared genomic backbone aimed at supporting fundamental metabolism, as well as partially sharing stress tolerance-related genes in *Chroococcidiopsis*, possibly bound to the desert ecological niches they are adapted to. This conservation is reflected in a high number of genes related to replication, recombination, and repair, as well as cell envelope biogenesis and energy metabolism, underscoring the centrality of genome maintenance and structural integrity under extreme conditions. Such a robust core likely represents an evolutionary adaptation to endure recurrent cycles of desiccation, irradiation, and metabolic arrest typical of lithic desert niches (Xu et al. 2021), which is corroborated by the absence of genes for the production of protective osmolites such as glucosylglycerol, typically found in marine cyanobacteria (Hagemann 2011).

### Compatible solutes and carbohydrate metabolism as central pillars of desiccation resistance

All three genomes encode a complete and conserved genetic repertoire for trehalose and sucrose biosynthesis, confirming the pivotal role of compatible solutes in desiccation tolerance (Billi and Potts 2002). Trehalose and sucrose are known to stabilize proteins, membranes, and nucleic acids during dehydration by replacing water molecules and promoting vitrification, a mechanism particularly relevant under prolonged space exposure (Crowe et al. 1992). Similarly, proline can act as a secondary compatible solute, enhancing osmotic stress resistance and thus desiccation tolerance (Klähn and Hagemann 2011). A previous *in-silico* survey of *Chroococcidiopsis* sp. CCMEE 029 identified pathways for trehalose and sucrose biosynthesis while gene overexpression and disaccharide accumulation were confirmed in response to desiccation. This adaptation strategy enabled cell viability after 7 years of air-dried storage as well as the survival of dried cells mixed with Mars regolith simulant and irradiated with 5.5 × 103 kJ/m^2^ of a Mars-like UV flux (Mosca et al. 2019). Although the CAZyme repertoires were largely conserved, subtle but meaningful differences emerged among strains, particularly in glycosyltransferase families involved in cell surface modification. The unique presence of GT27 enzymes and the reduced GT2_Glyco_transf_2 content in *Chroococcidiopsis* sp. CCMEE 064, suggests strain-specific modulation of protein and cell envelope glycosylation. Such variations may influence extracellular polymeric substance composition, surface charge, and interactions with mineral substrates, factors that are especially relevant for UV shielding, biofilm formation, and survival within lithic microhabitats (Kehr and Dittmann 2015). Notably, this strain was uniquely predicted to synthesize the nucleotide sugar UDP-QuiNAc, a key precursor for the biosynthesis of specialized cell surface glycoconjugates and lipopolysaccharide components (Páez-Watson et al. 2024), whose production may contribute to altered EPS composition or modified outer cell envelope structure. Indeed, in response to drying, *Chroococcidiopsis* has been reported to increase the envelope thickness and accumulate acid, sulphated and beta-linked polysaccharides, positively charged glycoproteins as well as lipids and proteins, that confer a resting-like morphology to the dried cells (Caiola et al. 1996).

### Extensive DNA protection and repair systems support long-term genomic integrity

Desiccation and radiation induce overlapping forms of DNA damage caused by reactive oxygen species (Mattimore and Battista 1996), including strand breaks and base modifications, making DNA protection and repair capacity a critical determinant of survival. The presence of multiple *dps* genes, including orthologs of NpDps proteins and bacterioferritin-like components, points to a multilayered DNA protection strategy combining physical shielding, iron sequestration, and oxidative damage mitigation (Li et al. 2018). Such redundancy likely enhances robustness during prolonged dormancy, when damage accumulation cannot be counteracted by active metabolism. Beyond protection, the identification of complete and overlapping DNA repair pathways (NER, BER, UVDE, MMR, and double-strand break repair) across all strains underscores a remarkable investment in genome maintenance. This broad repair repertoire aligns with previous observations that these desiccation-tolerant cyanobacteria rely on a synergy between damage avoidance and efficient DNA repair (Billi 2009), even though CCMEE 064 generally contained less genes in COG replication, repair and recombination category, which could be explained by a reduced genome size compared to CCMEE 029 and CCMEE 057. The conservation of these pathways across the investigated desert strains suggests that DNA integrity, upon desiccation and repair, likely function as prerequisites for coping with space and Mars-like conditions. Notably, genes encoding photolyase and enzymes of the nucleotide excision repair were up-regulated during the rewetting of *Chroococcidiopsis* sp. CCMEE 029 stored in the air-dried state for seven years and then exposed to 1.5 × 103 kJ/m^2^ of a Mars-like UV (Mosca et al. 2019). Similarly, the recombination RecF (present in two copies in CCMEE 029 in distal genomic loci) and base excision repair pathways were over-expressed during the rehydration of dried biofilms of *Chroococcidiopsis* sp. CCMEE 029 exposed to space-vacuum for 672 days during the BOSS space mission and responsible for repairing the accumulated double-strand breaks (Billi et al. 2019b, Mosca et al. 2021).

### Antioxidant diversity reflects both conserved defenses and strain-specific tuning

Reactive oxygen species represent a major threat during desiccation and irradiation, and all three *Chroococcidiopsis* genomes encode an extensive set of enzymatic and non-enzymatic antioxidant systems. The conserved presence of manganese- and copper/zinc-dependent superoxide dismutases, multiple catalases, peroxiredoxins, thioredoxins, and chaperones indicates a highly redundant redox-buffering network. Such redundancy likely ensures functional resilience when individual components are damaged or inactive during prolonged desiccation. At the same time, strain-specific differences in antioxidant gene copy numbers, like the exclusive presence of *sodB* in *Chroococcidiopsis* sp. CCMEE 064, the absence of *katG* in *Chroococcidiopsis* sp. CCMEE 057, and the expansion of glutathione-related genes and glyoxalase components in *Chroococcidiopsis* sp. CCMEE 029, points to a plethora of different strategies for ROS detoxification and stress management. The presence of multiple OCP-related carotenoid proteins, including an HCP4-like phycobilisome quencher, HCP2/HCP3-like singlet-oxygen quenchers, an HCP1-like paralog with likely auxiliary carotenoid-binding and stress-related roles, and CTDH as a carotenoid donor/loading factor further diversifies the non-enzymatic antioxidant inventory. Likewise, the four CAB/HLI loci encode small one-helix, light-harvesting-like proteins (Hlips), known to participate not only in photoprotection and energy quenching but also in chlorophyll biosynthesis and early photosystem assembly, thereby broadening the scope of “antioxidant” functions beyond classical ROS-scavenging enzymes (Komenda and Sobotka 2016, Sheppard et al. 2025, Wysocka et al. 2025). This finding is in agreement with the previous *in silico* analysis of CCMEE 029 that identified three superoxide dismutases, four catalases and five peroxidases, that were all upregulated after H_2_O_2_ treatment (Di Stefano et al. 2025). Indeed, differences may influence the balance between metal-dependent redox chemistry, peroxide detoxification routes and methylglyoxal metabolism, potentially affecting survival outcomes under different radiation spectra or oxidative regimes encountered in space and Mars-like environments.

### Photoprotective secondary metabolites and space survival

The discovery of a conserved and syntenic scytonemin BGC across all three strains reinforces the importance of this pigment as a conserved photoprotective strategy in *Chroococcidiopsis*. Scytonemin’s ability to absorb UVA and UVB radiation (Gao and Garcia-Pichel 2011) makes it particularly relevant under extraterrestrial conditions characterized by intense and unfiltered solar radiation. The conservation of export-related genes further suggests that efficient localization of scytonemin to the extracellular matrix is a key adaptive feature (Parrett et al. 2025). The exclusive presence of the mycosporine-glycine biosynthetic genes in *Chroococcidiopsis* sp. CCMEE 057 adds an additional layer of photoprotection, potentially conferring enhanced tolerance to specific UV regimes, even in the dried, ametabolic state. The physical linkage between MAAs and scytonemin clusters in this strain hints at coordinated regulation and evolutionary coupling of UV defense mechanisms, possibly reflecting adaptation to microenvironments with distinct spectral irradiation profiles. Taken together, these findings support a model in which the extraordinary resilience of dried *Chroococcidiopsis* to space and Mars-like conditions arises from the interplay between a highly conserved stress tolerance core and flexible, strain-specific adaptations. Rather than relying on singular “extreme” traits, these cyanobacteria seem to deploy redundant, overlapping protective systems that ensure survival during prolonged periods of metabolic inactivity and facilitate recovery upon rehydration.

### Implications for applied astrobiology

The genomic features identified in this study strengthen the relevance of desert strains of *Chroococcidiopsis* not only as model organisms for exploring the limits of life and its search beyond Earth, but also to develop technologies to support human outposts in space, which might not be regarded as separate challenges (Wordsworth et al. 2025). Indeed the robustness of DNA repair upon reactivation after space exposure of *Chroococcidiopsis* sp. CCMEE 029 (Napoli et al. 2022) not only expanded our knowledge on the endurance potential of life as we know it, but provided also a significant step-change in the employment of this cyanobacterium in space bioprocesses, since it supports space travel in the dormant state and rehydration at human outposts on the Moon (or Mars). Moreover, the combination of photoprotection, antioxidant robustness, and metabolic minimalism positions *Chroococcidiopsis* strains as promising candidates for bioregenerative life support systems (BLSS) and *in situ* resource utilization strategies (ISRU). From an ISRU perspective, previous studies have shown that *Chroococcidiopsis* sp. CCMEE 029 is capable of growing when mixed with perchlorate-rich Mars regolith simulants supplemented with synthetic urine and has been successfully employed to produce biomass as feedstock for bacteria (Fernandez et al. 2023). Furthermore, the recognition of this strain formaldehyde assimilation potential represented by the possibility to employ the RuMP pathway and the biosynthetic capacity to produce tocopherol make it a highly valuable subject for ISRU (Averesch 2021, Rigano et al. 2026). Similarly, *Chroococcidiopsis* sp. CCMEE 057 could be a promising candidate to produce MAAs and scytonemin, implying its use as a cell factory as well if production is confirmed. In conclusion, future experiments are needed to open new frontiers towards biotechnological development of *Chroococcidiopsis* strains to support human space exploration.

## Supplementary Material

xtag036_Supplemental_Files
