# Supplementary figures and images for: Comparative genomics of space-resilient Chroococcidiopsis cyanobacteria reveals a core genetic repertoire supporting extreme tolerance towards desert and non-Earth conditions
Source: FEMS Microbes. 2026 Jun 13;7:xtag036. doi: 10.1093/femsmc/xtag036 (PMC13308351; doi:10.1093/femsmc/xtag036)

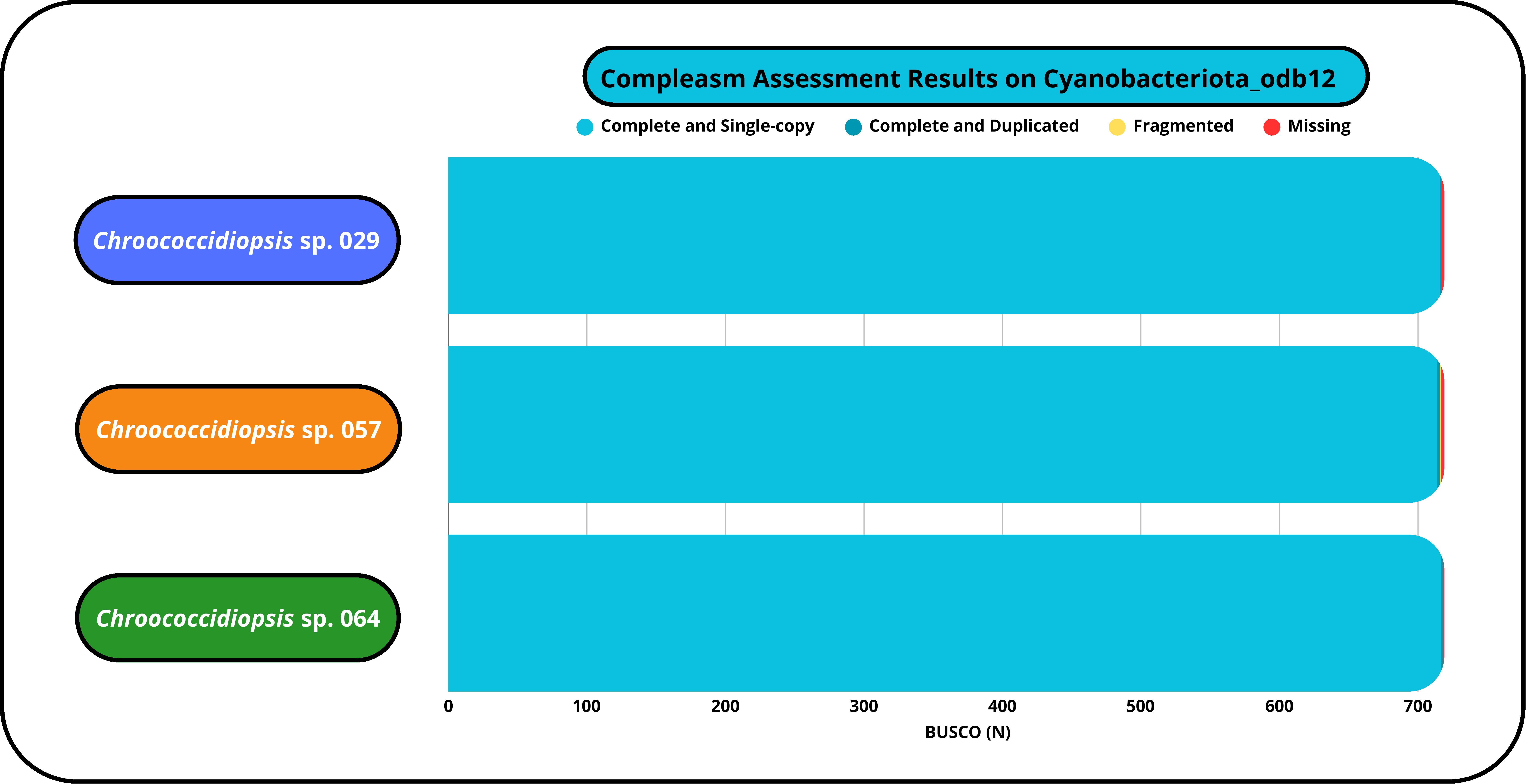

Supplement: xtag036_Supplemental_Files [file xtag036_supplemental_files.zip › Supplementary_Figure.jpg]
