# Supplementary material for: Comparative genomics of space-resilient Chroococcidiopsis cyanobacteria reveals a core genetic repertoire supporting extreme tolerance towards desert and non-Earth conditions
Source: FEMS Microbes. 2026 Jun 13;7:xtag036. doi: 10.1093/femsmc/xtag036 (PMC13308351; doi:10.1093/femsmc/xtag036)
Supplement: xtag036_Supplemental_Files [file xtag036_supplemental_files.zip › FEMSMC-2026-020.R1 supplementary data - reviews and author response.docx]

**Reviews of manuscript FEMSMC-2026-020**

**Reviewer 1: Igor Pessi, University of Helsinki**

**Recommendation: Major Revision**

Rigano et al. describes the genomes of three Chroococcidiopsis strains with a focus on the genomic features that enable their survival in space- and Mars-like conditions. This is an interesting manuscript but contains several issues that need to be addressed.

The study is quite descriptive and not hypothesis-driven which, in my opinion, is not a bad thing. However, instead of embracing this reality, the authors go to great lengths to try to oversell the impact of their work beyond reasonability, including the use of exaggerated adjectives that are not on par with the scale of the study. For example, the authors claim to undertake “a comprehensive comparative genome analysis” to “investigate in detail the genetic determinants” associated with desiccation resistance and other environmental stresses. I don’t think that the experimental design (n = 3 genomes) allows this. There are dozens of genomes of Chroococcidiopsis in GenBank that could have been included to fortify the experimental design, particularly if genomes from other environments would have been included. If a “comprehensive” analysis was intended, why only three genomes were selected? In the end, the authors have sequenced two novel genomes of Chroococcidiopsis, which is a valuable resource for the scientific community, but the novelty of the work beyond this is not clear. This issue is evident on the Discussion, which reads as a long literature review of microbial adaptation to environmental stresses without clear indication of the specific contributions of the present work. I strongly suggest the authors to rethink their strategy, either by implementing a more adequate experimental design or reframing/streamlining the manuscript along the lines of a “genome announcement” type of article.

Abstract

- “how they can recover [..] remains to be unveiled”: this is an example of an exaggerated tone which gives the impression that nothing is known on the topic. In fact, a lot is known about how Chroococcidiopsis sustain harsh environmental conditions. What is the specific issue that the authors are referring to?

- “that survived [..] space exposure and Mars-like conditions”: it can be understood by this as if the populations that were sequenced were the same as those that survived in space, like it was done for the strain CCMEE 029 in Napoli et al. (2022). This does not seem to be the case in this study.

- “unique genetic elements”: what does this mean?

Introduction

- “How desert strains of Chroococcidiopsis [..] are able to recover [..] remains to be unveiled”: this is too vague; what “remains to be unveiled”, specifically?

- “we present a comprehensive comparative genome analysis”: I would suggest toning down sentences like this. Of course, comprehensiveness is something that is difficult to quantify, but I think using this adjective in the context of the present study, which analysed only three genomes, is pushing semantics a bit too far.

- “targeted genome mining”: what does this mean? How is this different from what is described in the previous sentence?

Materials and Methods

- “Chroococcidiopsis sp. CCMEE 057 and CCMEE 064 were isolated” and “These strains are maintained”: please add the isolation dates and how the cultures have been maintained since then (cryopreservation, sequential culturing).

- “Draft genome assemblies were generated with Flye v2.9.2-b1786”: please clarify if hybrid or Nanopore-only assemblies were used.

Results

- “A single circular chromosome-level assembly was obtained”: how was this was assessed?

- “The comparative analysis [..] revealed a highly conserved core”: what does this mean?

Discussion

- “In this study we provide [..]”: this paragraph says a lot but it does not say anything in fact; what is the actual contribution of this study? In addition, please reconsider the use of words like “comprehensive”, “decipher”, “remarkable” and “disentangle”, which read a bit over-the-top.

- “The identification of a large conserved core proteome [..] highlights the existence of a shared genomic backbone supporting fundamental stress tolerance mechanisms in Chroococcidiopsis”: I completely disagree with this statement. It is likely that a huge part of this core genome is actually housekeeping genes that are found in other organisms and have nothing to do with stress tolerance.

- “enrichment of COG categories”: enriched compared to what?

Figures

- Fig. 1 could work better as a table.

- Fig. 3 has too many colours that are difficult to differentiate.

**Reviewer 2: Amit Srivastava, Purdue University**

**Recommendation: Major Revision**

This manuscript reports a comparative genomic analysis of three desert Chroococcidiopsis strains , CCMEE 029, 057, and 064 , that have survived space- and Mars-like conditions in prior experiments. The work is timely, the organism is a well-established astrobiology model, and the analytical scope is appropriate. New chromosome-level assemblies are generated for two strains (CCMEE 057 and 064), a pangenome analysis is performed, and stress-tolerance gene families are systematically surveyed. These are genuine contributions.

That said, the manuscript has several important weaknesses: key genomic comparisons lack supporting statistics and quality metrics; functional conclusions frequently go further than the data allow. The revisions requested below are substantial but addressable, and the work has real potential once they are made.

1. In the antioxidants Results section, the authors write that the non-enzymatic antioxidant reservoir is "particularly rich" in OCP genes, with five copies found in all three strains. Having multiple ocp paralogs is not remarkable for cyanobacteria of this genome size. Importantly, the five genes almost certainly belong to distinct OCP subfamilies , OCP1, OCP2, OCPX, and RCP-like variants , which have very different functions and interaction partners. Lumping them together as "five OCP genes" obscures this. The authors should identify which subfamilies are present and discuss their specific roles. Supplementary Table 1 currently does not list the OCP gene IDs, making verification impossible. Key references to consult: Melnicki et al. 2016 (Plant Cell); Bao et al. 2017 (Nat Commun). The text states OCP is "fundamental for photosynthetic apparatus integrity through non-photochemical quenching." This is broadly correct, but OCP-driven NPQ works by the active (red) form binding to the phycobilisome after light-induced carotenoid translocation within the protein. This process requires active metabolism and light. The authors do not explain how OCP is relevant in dried, ametabolic cells , the very state being studied. They should either explain the connection (e.g., OCP expression and loading before desiccation confers protection upon rehydration) or qualify the statement as a mechanism relevant only when cells are hydrated and active. The "four copies" of CAB/HLI proteins include one-helix proteins (OHPs/HLIPs) whose roles extend well beyond light harvesting , they are involved in chlorophyll biosynthesis and photoprotection via distinct mechanisms. Grouping them as a single category without comment understates the functional diversity.

2. With only three genomes, formal statistical testing is limited , but the following are achievable and expected. Average Nucleotide Identity (ANI) between the three strains is never calculated. Without this, readers cannot judge whether the strains are genuinely divergent (making comparative genomics informative) or so similar that observed copy-number differences are within annotation noise. Please add an ANI matrix , this is a standard, quick calculation. Differences like "five glutathione reductase copies in CCMEE 057" or "almost double the GST genes in CCMEE 029" are presented as biological facts, but copy number inflation can easily arise from assembly artifacts or overly permissive annotation. The authors should verify these differences by checking, for example, whether the duplicated genes sit in different genomic locations and have distinct sequences, rather than being annotation artifacts from repeated regions. The pangenome core/accessory/singleton partitioning (80.2% core) depends heavily on the identity and coverage thresholds used in RIBAP. These must be reported, and the resulting core fraction should be compared to what is expected for strains at this divergence level.

3. BUSCO completeness (>99%) is reported, which is good. However, several standard quality metrics are absent. N50, total contig number before and after polishing, and whether the assemblies are truly circular or contain unresolved gaps should all be stated. The claim of "single circular chromosome-level assembly" needs to be substantiated. For Pilon polishing (five iterations), the number of Illumina reads and mean depth of coverage are essential for readers to judge the reliability of variant calls and copy-number estimates. PROKKA, EggNOG-mapper, and InterProScan are all applied. It is unclear which tool's gene calls form the master annotation, and how conflicts between tools were resolved. Please describe the pipeline explicitly. This is currently mentioned only briefly in Methods. It should be stated clearly in the Introduction or Results, and the quality of the older sequencing library relative to the newly generated ones should be assessed and discussed.

4. Gene presence in a genome does not equal gene function or expression. Several passages make this leap without adequate qualification. The text mentions that CCMEE 029 uniquely carries the RuMP (ribulose monophosphate) pathway for formaldehyde assimilation, and then cites Fernandez et al. 2023 to note the strain can grow in perchlorate-rich Mars regolith simulants. These two facts are placed together in a way that implies the RuMP pathway explains the perchlorate growth , but the RuMP pathway has nothing to do with perchlorate metabolism. Either explain the actual connection or decouple these two observations. The authors note that CCMEE 057 lacks the catalase-peroxidase gene katG, which is a significant difference. But they do not ask the obvious question: does it matter? CCMEE 057 has five glutathione reductase copies and an expanded peroxiredoxin set that may fully compensate for the loss. The Discussion should address whether katG absence is likely to have functional consequences or whether it is buffered by redundancy. The suggestion that CCMEE 057 "could be a perfect candidate" for producing MAAs and scytonemin as a biotechnology platform is speculative. The BGC genes are present, but without any transcriptomic, proteomic, or metabolomic evidence that these clusters are actually expressed under relevant conditions, this remains a hypothesis. Please frame it accordingly.

5. The manuscript compares only three strains of one genus with no reference to wider Chroococcidiopsis diversity. Other genome sequences are publicly available (e.g., CCMEE 171, C. thermalis PCC 7203). Without including these, or at minimum providing a whole-genome phylogeny or ANI matrix that positions the three study strains within the genus, it is impossible to assess whether the observed core/accessory genome structure is typical for Chroococcidiopsis or is simply a result of comparing three closely related strains. This context is essential for interpreting the pangenome results.

6. The abstract currently reads as two independent summaries joined together , one reporting results, one discussing implications , without a clear narrative flow. Revise to lead with the biological question, briefly state the approach, summarise the key findings, and end with significance.

7. The abstract mentions the BGC findings but does not state that new chromosome-level genome assemblies were generated for CCMEE 057 and 064. This is a core deliverable of the study and should appear in the abstract.

8. The three aims listed in the final paragraph of the Introduction are redundant , aims (i) and (ii) are effectively subsets of aim (iii). Streamline to two clearly distinct aims.

9. This section reads more like a literature review of space biotechnology possibilities than a focused discussion of what this study specifically shows. Consider shortening it significantly and redirecting the space to a more mechanistic discussion of what the genomic comparisons actually reveal.

10. The Discussion does not clearly separate what is genuinely new (chromosome-level resolution, two new assemblies, strain-specific gene differences) from what confirms prior in-silico analyses (e.g., Napoli et al. 2022; Di Stefano et al. 2025). This distinction matters for readers and for the journal.

11. The Introduction cites Cox & Battista 2005 (Nature Reviews Microbiology) when describing Chroococcidiopsis as radiation-tolerant. That review is primarily about Deinococcus radiodurans, not Chroococcidiopsis. A primary reference specifically documenting Chroococcidiopsis radiation tolerance should be used here.

12. "Billi 2026" needs a DOI or "in press" label. This reference is cited twice but no volume, page, or DOI is given. If the article has been published, please provide the full citation. If still in press, label it clearly.

13. "UVER" (UvsE-dependent Excision Repair) is not standard terminology that most readers will know. Introduce it with a brief explanation and cite the relevant primary literature.

14. The most abundant COG category is L (replication, recombination and repair), with 580, 557, and 329 genes for strains 029, 057, and 064 respectively. The nearly 2-fold difference between CCMEE 029 and CCMEE 064 is never discussed. Is this due to the larger genome size of 029? A genuine difference in repair gene content? Or an annotation artifact? This should be addressed.

15. The text states "60 out of 65 complete modules were shared" but only discusses the strain-specific absent modules. Which 5 modules are absent across all three strains (or in more than one strain)? These should be listed explicitly.

16. Sucrose synthase (susA) appears twice in Supplementary Table 1 (groups 1142 and 2112) in all three strains. Are these paralogs with different expression patterns, or recent duplications? A one-sentence explanation is needed.

17. The gene is described as "possibly acting as a dual-functioning sucrose synthase and phosphatase." Is this a bioinformatic prediction or has it been demonstrated biochemically? Make the level of evidence explicit , readers cannot act on "possibly."

18. The listing of adk, gmk, tmk, ndk, etc. under DNA repair-related genes is incorrect. These are nucleotide metabolism enzymes, not repair factors. They should either be moved to the metabolic section or their indirect relevance to DNA repair (e.g., maintaining dNTP pools) should be explicitly explained.

19. CCMEE 029 has two recF orthologs (groups 4008 and 1013), whereas the other strains have only one. This is unusual and potentially interesting , is the extra copy in a genomic island? Is it a recent duplication? Even a brief note in the Discussion would be informative.

20. Genes such as recD2 and recF are listed as NA for CCMEE 057 and 064 in the HR section. "NA" could mean genuinely absent, not detected, or not annotated by any tool used. Please clarify , this distinction is biologically critical.

21. The authors note the absence of ggpP/ggpS and gsmT/sdmT genes. This is ecologically interesting because these are osmolytes typical of marine/brackish cyanobacteria, not terrestrial ones. A brief sentence connecting their absence to the desert-adapted lifestyle would strengthen the ecological framing.

22. Figure 5 colour scale is dominated by one category. The heatmap colour scale (0–21) is dominated by the HR category (21, 20, 19 genes), which means most other categories appear in nearly the same low-intensity colour, making differences hard to see. Consider either using a log scale or displaying each category row with its own normalised scale.

23. Figure 6 legend is incomplete. The synteny figure uses several colour-coded modules but the legend does not define all of them clearly. Each colour/module should be explicitly described. Additionally, the statement that the MAA cluster "directly preceded" the scytonemin cluster in CCMEE 057 should include the inter-cluster distance in base pairs, and any evidence for co-regulation (shared promoters, regulatory genes) should be mentioned or its absence noted.

24. Typo in CAZyme family name. Supplementary Table 2 uses "GT2_Glyco_tranf_2" (missing an 's'), while the main text uses "GT2_Glyco_transf_2." Please standardise.

**Associate Editor Recommendation: Major Revision**

Dear Professor Billi,

Thank you for submitting your manuscript:

"Comparative genomics of space-resilient Chroococcidiopsis cyanobacteria reveals a core genetic repertoire supporting extreme tolerance towards desert and non-Earth conditions"

I'm pleased to let you know that your work has now been evaluated by two peer reviewers, and I'm glad to share that both of them recognised the interest and relevance of your study .

Following their careful review, both referees recommend major revisions prior to acceptance. While they have identified some substantial points to address, they are confident these are fully manageable. The main issues raised concern the lack of statistical details for the draft assemblies, and the number of Chroococcidiopsis genomes included in the comparative analysis.

I warmly encourage you to take their feedback on board and resubmit a revised version of your manuscript. I have no doubt that addressing these points will further strengthen what is already a very promising piece of work.

Please don't hesitate to reach out if you have any questions along the way — we're here to support you through the process.

With kind regards,

Yannick Lara

**EIC Decision: Major Revision**

**Author's Response to Decision Letter for (FEMSMC-2026-020)**

We thank the reviewers for the precious advice and comments that they provided. This helped us to address important aspects of our research and contributed to give it a better scientific impact.

Reviewer: 1

Comments to the Author

Rigano et al. describes the genomes of three Chroococcidiopsis strains with a focus on the genomic features that enable their survival in space- and Mars-like conditions. This is an interesting manuscript but contains several issues that need to be addressed.

The study is quite descriptive and not hypothesis-driven which, in my opinion, is not a bad thing. However, instead of embracing this reality, the authors go to great lengths to try to oversell the impact of their work beyond reasonability, including the use of exaggerated adjectives that are not on par with the scale of the study. For example, the authors claim to undertake “a comprehensive comparative genome analysis” to “investigate in detail the genetic determinants” associated with desiccation resistance and other environmental stresses. I don’t think that the experimental design (n = 3 genomes) allows this. There are dozens of genomes of Chroococcidiopsis in GenBank that could have been included to fortify the experimental design, particularly if genomes from other environments would have been included. If a “comprehensive” analysis was intended, why only three genomes were selected? In the end, the authors have sequenced two novel genomes of Chroococcidiopsis, which is a valuable resource for the scientific community, but the novelty of the work beyond this is not clear. This issue is evident on the Discussion, which reads as a long literature review of microbial adaptation to environmental stresses without clear indication of the specific contributions of the present work. I strongly suggest the authors to rethink their strategy, either by implementing a more adequate experimental design or reframing/streamlining the manuscript along the lines of a “genome announcement” type of article.

Abstract

- “how they can recover [..] remains to be unveiled”: this is an example of an exaggerated tone which gives the impression that nothing is known on the topic. In fact, a lot is known about how Chroococcidiopsis sustain harsh environmental conditions. What is the specific issue that the authors are referring to?

A: It has now been reframed as: However, how they can recover upon rehydration and repair the damage accumulated under extreme conditions on Earth and in space remains to be fully elucidated through omics-based investigations

- “that survived [..] space exposure and Mars-like conditions”: it can be understood by this as if the populations that were sequenced were the same as those that survived in space, like it was done for the strain CCMEE 029 in Napoli et al. (2022). This does not seem to be the case in this study.

A: It has now been reframed as: The analysis of newly obtained gapless genome assemblies of the laboratory-maintained reference strains of strains CCMEE 029, 057 and 064 allowed the identification of conserved and non-shared genes.

- “unique genetic elements”: what does this mean?

A: It has now been reframed as “non-shared genes”.

Introduction

- “How desert strains of Chroococcidiopsis [..] are able to recover [..] remains to be unveiled”: this is too vague; what “remains to be unveiled”, specifically?

A: It has now been reframed as: However, how they can recover upon rehydration and repair the damage accumulated under extreme conditions on Earth and in space remains to be fully elucidated through omics-based investigations

- “we present a comprehensive comparative genome analysis”: I would suggest toning down sentences like this. Of course, comprehensiveness is something that is difficult to quantify, but I think using this adjective in the context of the present study, which analysed only three genomes, is pushing semantics a bit too far.

A: This has now been reframed as “comparative genome analysis of three space-resistant Chroococcidiopsis strains”.

- “targeted genome mining”: what does this mean? How is this different from what is described in the previous sentence?

A: Many softwares like antiSMASH fail to annotate some slightly divergent biosynthetic gene clusters compared to its database, hence a targeted genome mining strategy (manual) which allowed us to find genome clusters for scytonemins and MAAs.

Materials and Methods

- “Chroococcidiopsis sp. CCMEE 057 and CCMEE 064 were isolated” and “These strains are maintained”: please add the isolation dates and how the cultures have been maintained since then (cryopreservation, sequential culturing).

A: Chroococcidiopsis sp. CCMEE 057 and CCMEE 064 were collected in 1969 by E. Imre Friedmann from chasmoendolithic and hypolithic communities in the Sinai Desert respectively and isolated by Roseli Ocampo-Friedmann from chasmoendolithic and hypolithic communities in the Sinai Desert respectively. While Chroococcidiopsis sp. CCMEE 029 was collected in 1969 by E. Imre Friedmann in 1969 from cryptoendolithic communities in the Negev desert and isolated by Roseli Ocampo-Friedmann. Starting from 2006 tThese strains are maintained through sequential culturing at the University of Rome Tor Vergata ..

- “Draft genome assemblies were generated with Flye v2.9.2-b1786”: please clarify if hybrid or Nanopore-only assemblies were used.

A: It has now been clarified “using Nanopore reads”. Illumina data were only used for genome assembly polishing.

Results

- “A single circular chromosome-level assembly was obtained”: how was this was assessed?

A: This has now been addressed “Genome assembly contiguity was assessed through manual inspection with Bandage v0.9 (Wick et al. 2015).”

- “The comparative analysis [..] revealed a highly conserved core”: what does this mean?

A: This has been reframed as “The comparative analysis on protein-coding genes revealed a high number of shared genes, making up a core proteome of “

Discussion

- “In this study we provide [..]”: this paragraph says a lot but it does not say anything in fact; what is the actual contribution of this study? In addition, please reconsider the use of words like “comprehensive”, “decipher”, “remarkable” and “disentangle”, which read a bit over-the-top.

- “The identification of a large conserved core proteome [..] highlights the existence of a shared genomic backbone supporting fundamental stress tolerance mechanisms in Chroococcidiopsis”: I completely disagree with this statement. It is likely that a huge part of this core genome is actually housekeeping genes that are found in other organisms and have nothing to do with stress tolerance.

A: This has been reframed more correctly as “The identification of a large conserved core proteome, accounting for over 80% of the protein-coding genes across the three strains, highlights the existence of a shared genomic backbone aimed at supporting fundamental metabolism, as well as partially sharing stress tolerance-related genes in Chroococcidiopsis, possibly bound to the desert ecological niches they are adapted to. This conservation is reflected in a high number of genes related to replication, recombination, and repair, as well as cell envelope biogenesis and energy metabolism, underscoring the centrality of genome maintenance and structural integrity under extreme conditions.”

- “enrichment of COG categories”: enriched compared to what?

A: Reframed as “in a high number of genes related to”

Figures

- Fig. 1 could work better as a table.

A: Figure 1 has been moved to supplementary Figure 1

- Fig. 3 has too many colours that are difficult to differentiate.

A: Figure 3 has been simplified

Reviewer: 2

Comments to the Author

This manuscript reports a comparative genomic analysis of three desert Chroococcidiopsis strains , CCMEE 029, 057, and 064 , that have survived space- and Mars-like conditions in prior experiments. The work is timely, the organism is a well-established astrobiology model, and the analytical scope is appropriate. New chromosome-level assemblies are generated for two strains (CCMEE 057 and 064), a pangenome analysis is performed, and stress-tolerance gene families are systematically surveyed. These are genuine contributions.

That said, the manuscript has several important weaknesses: key genomic comparisons lack supporting statistics and quality metrics; functional conclusions frequently go further than the data allow. The revisions requested below are substantial but addressable, and the work has real potential once they are made.

1. In the antioxidants Results section, the authors write that the non-enzymatic antioxidant reservoir is "particularly rich" in OCP genes, with five copies found in all three strains. Having multiple ocp paralogs is not remarkable for cyanobacteria of this genome size. Importantly, the five genes almost certainly belong to distinct OCP subfamilies , OCP1, OCP2, OCPX, and RCP-like variants , which have very different functions and interaction partners. Lumping them together as "five OCP genes" obscures this. The authors should identify which subfamilies are present and discuss their specific roles. Supplementary Table 1 currently does not list the OCP gene IDs, making verification impossible. Key references to consult: Melnicki et al. 2016 (Plant Cell); Bao et al. 2017 (Nat Commun). The text states OCP is "fundamental for photosynthetic apparatus integrity through non-photochemical quenching." This is broadly correct, but OCP-driven NPQ works by the active (red) form binding to the phycobilisome after light-induced carotenoid translocation within the protein. This process requires active metabolism and light. The authors do not explain how OCP is relevant in dried, ametabolic cells, the very state being studied. They should either explain the connection (e.g., OCP expression and loading before desiccation confers protection upon rehydration) or qualify the statement as a mechanism relevant only when cells are hydrated and active. The "four copies" of CAB/HLI proteins include one-helix proteins (OHPs/HLIPs) whose roles extend well beyond light harvesting, they are involved in chlorophyll biosynthesis and photoprotection via distinct mechanisms. Grouping them as a single category without comment understates the functional diversity.

A: An OCP and CAB/HLI characterization has now been conducted and discussed in the text.

2. With only three genomes, formal statistical testing is limited , but the following are achievable and expected. Average Nucleotide Identity (ANI) between the three strains is never calculated. Without this, readers cannot judge whether the strains are genuinely divergent (making comparative genomics informative) or so similar that observed copy-number differences are within annotation noise. Please add an ANI matrix , this is a standard, quick calculation. Differences like "five glutathione reductase copies in CCMEE 057" or "almost double the GST genes in CCMEE 029" are presented as biological facts, but copy number inflation can easily arise from assembly artifacts or overly permissive annotation. The authors should verify these differences by checking, for example, whether the duplicated genes sit in different genomic locations and have distinct sequences, rather than being annotation artifacts from repeated regions. The pangenome core/accessory/singleton partitioning (80.2% core) depends heavily on the identity and coverage thresholds used in RIBAP. These must be reported, and the resulting core fraction should be compared to what is expected for strains at this divergence level.

A: ANI matrix and whole-genome based phylogenetic analysis on available Chroococcidiopsis genomes has been carried out and added to the manuscript. The multiple-copy genes are not annotation artifacts, as the gene ID numbering follows gene contiguity, hence pointing out to different gene loci throughout the genome. The core/accessory/singleton calculation , run with OrthoVenn3 (which uses Orthofinder) was run with default parameters. This specification has now been expanded in the Materials and Methods section.

3. BUSCO completeness (>99%) is reported, which is good. However, several standard quality metrics are absent. N50, total contig number before and after polishing, and whether the assemblies are truly circular or contain unresolved gaps should all be stated. The claim of "single circular chromosome-level assembly" needs to be substantiated. For Pilon polishing (five iterations), the number of Illumina reads and mean depth of coverage are essential for readers to judge the reliability of variant calls and copy-number estimates. PROKKA, EggNOG-mapper, and InterProScan are all applied. It is unclear which tool's gene calls form the master annotation, and how conflicts between tools were resolved. Please describe the pipeline explicitly. This is currently mentioned only briefly in Methods. It should be stated clearly in the Introduction or Results, and the quality of the older sequencing library relative to the newly generated ones should be assessed and discussed.

A: Since the genome assemblies are gapless, circular and single chromosome the N50 statistic would have been meaningless, as the pre-polishing/post-polishing results from Pilon, which does not scaffold any sequences, but rather fix small assembly breaks and erroneous bases. Nonetheless, we now sustain the claim "single circular chromosome-level assembly" with Bandage manual visualization and quast statistics. The annotation pipeline is now described more thoroughly. Regarding the sequencing data, they had all been sequenced roughly at the same time with the same chemistry, hence there is no particular difference in sequencing.

4. Gene presence in a genome does not equal gene function or expression. Several passages make this leap without adequate qualification. The text mentions that CCMEE 029 uniquely carries the RuMP (ribulose monophosphate) pathway for formaldehyde assimilation, and then cites Fernandez et al. 2023 to note the strain can grow in perchlorate-rich Mars regolith simulants. These two facts are placed together in a way that implies the RuMP pathway explains the perchlorate growth , but the RuMP pathway has nothing to do with perchlorate metabolism. Either explain the actual connection or decouple these two observations. The authors note that CCMEE 057 lacks the catalase-peroxidase gene katG, which is a significant difference. But they do not ask the obvious question: does it matter? CCMEE 057 has five glutathione reductase copies and an expanded peroxiredoxin set that may fully compensate for the loss. The Discussion should address whether katG absence is likely to have functional consequences or whether it is buffered by redundancy. The suggestion that CCMEE 057 "could be a perfect candidate" for producing MAAs and scytonemin as a biotechnology platform is speculative. The BGC genes are present, but without any transcriptomic, proteomic, or metabolomic evidence that these clusters are actually expressed under relevant conditions, this remains a hypothesis. Please frame it accordingly.

A: A new citation with formaldehyde assimilation linked to perchlorates has been added (Rigano et al. 2026); MAAs and scytonemin production capability from Chroococcidiopsis sp. 057 has been reframed and toned down. The discussion section addressed the katG gene presence/abscence.

5. The manuscript compares only three strains of one genus with no reference to wider Chroococcidiopsis diversity. Other genome sequences are publicly available (e.g., CCMEE 171, C. thermalis PCC 7203). Without including these, or at minimum providing a whole-genome phylogeny or ANI matrix that positions the three study strains within the genus, it is impossible to assess whether the observed core/accessory genome structure is typical for Chroococcidiopsis or is simply a result of comparing three closely related strains. This context is essential for interpreting the pangenome results.

A: A phylogenetic tree based on all the genome assemblies available from the NCBI database has been produced and an ANI matrix for the three investigated strains has been added.

6. The abstract currently reads as two independent summaries joined together , one reporting results, one discussing implications , without a clear narrative flow. Revise to lead with the biological question, briefly state the approach, summarise the key findings, and end with significance.

A: The abstract has been extensively rephased.

7. The abstract mentions the BGC findings but does not state that new chromosome-level genome assemblies were generated for CCMEE 057 and 064. This is a core deliverable of the study and should appear in the abstract.

A: This has now been added to the abstract.

8. The three aims listed in the final paragraph of the Introduction are redundant , aims (i) and (ii) are effectively subsets of aim (iii). Streamline to two clearly distinct aims.

A: It has now been reframed as: In this study, a comparative genome analysis of the three experimentally tested space-resistant Chroococcidiopsis sp. CCMEE 029, 057, and 064 was performed by integrating gene content analysis, functional annotation and metabolic pathway reconstruction to investigate the core and accessory proteomes associated to desiccation resistance, DNA damage protection and repair, oxidative stress mitigation.

9. This section reads more like a literature review of space biotechnology possibilities than a focused discussion of what this study specifically shows. Consider shortening it significantly and redirecting the space to a more mechanistic discussion of what the genomic comparisons actually reveal.

A: This section has been re-written.

10. The Discussion does not clearly separate what is genuinely new (chromosome-level resolution, two new assemblies, strain-specific gene differences) from what confirms prior in-silico analyses (e.g., Napoli et al. 2022; Di Stefano et al. 2025). This distinction matters for readers and for the journal.

A: The work by Napoli et al was reported as follows:. Indeed the robustness of DNA repair upon reactivation after space exposure of Chroococcidiopsis sp.CCMEE 029 (Napoli et al. 2022) not only expanded our knowledge on the endurance potential of life as we know it, but provided also a significant step-change the employment of this cyanobacterium in space bioprocesses since it supports space travel in the dormant state and rehydration at human outposts on the Moon (or Mars).

This sentence was added “This finding is in agreement with the previous in-silico analysis of CCMEE 029 that identified three superoxide dismutases, four catalases and five peroxidases, that were all upregulated after H2O2 treatment (Di Stefano et al. 2025).

11. The Introduction cites Cox & Battista 2005 (Nature Reviews Microbiology) when describing Chroococcidiopsis as radiation-tolerant. That review is primarily about Deinococcus radiodurans, not Chroococcidiopsis. A primary reference specifically documenting Chroococcidiopsis radiation tolerance should be used here.

A: The paper by Billi et al. 2000 “Ionizing-Radiation Resistance in the Desiccation-Tolerant Cyanobacterium Chroococcidiopsis” as been added and the review by Cox and Battista 2005 was maintained since in Table 2 it compares Chroococcidiopsis’s radioresistance with that of other radioresistant bacteria.

12. "Billi 2026" needs a DOI or "in press" label. This reference is cited twice but no volume, page, or DOI is given. If the article has been published, please provide the full citation. If still in press, label it clearly.

A: DOI added. “Billi D. Desert cyanobacteria under non-Earth conditions: Implications for astrobiology and sustainable life support. Acta Astronautica 2026;238:209–14. https://doi.org/10.1016/j.actaastro.2025.09.022.”

13. "UVER" (UvsE-dependent Excision Repair) is not standard terminology that most readers will know. Introduce it with a brief explanation and cite the relevant primary literature.

A: It has now been reframed as: the UVDE-mediated excision repair (Goosen and Moolenaar 2008), together with other genes encoding DNA repair proteins like radC and nucleoside kinases (adk, gmk, tmk, ndk, etc.; Fig. 5).

14. The most abundant COG category is L (replication, recombination and repair), with 580, 557, and 329 genes for strains 029, 057, and 064 respectively. The nearly 2-fold difference between CCMEE 029 and CCMEE 064 is never discussed. Is this due to the larger genome size of 029? A genuine difference in repair gene content? Or an annotation artifact? This should be addressed.

A: This is now addressed in the manuscript in the discussion section : “even though CCMEE 064 generally contained less genes in COG replication, repair and recombination category, which could be explained by a reduced genome size compared to CCMEE 029 and CCMEE 057”

15. The text states "60 out of 65 complete modules were shared" but only discusses the strain-specific absent modules. Which 5 modules are absent across all three strains (or in more than one strain)? These should be listed explicitly.

A: Figure 4 lists all shared and non shared complete modules. The 5 modules that are exclusively found/not found in the strains are already covered and discussed in the manuscript: RuMP-PPP-archeal pathway, UDP-QuiNAc, Tocopherol, CMP-KDO.

16. Sucrose synthase (susA) appears twice in Supplementary Table 1 (groups 1142 and 2112) in all three strains. Are these paralogs with different expression patterns, or recent duplications? A one-sentence explanation is needed.

A: This has now been addressed in the results section : “in distal parts of their genomes”.

17. The gene is described as "possibly acting as a dual-functioning sucrose synthase and phosphatase." Is this a bioinformatic prediction or has it been demonstrated biochemically? Make the level of evidence explicit , readers cannot act on "possibly."

A: This has now been addressed more specifically.

18. The listing of adk, gmk, tmk, ndk, etc. under DNA repair-related genes is incorrect. These are nucleotide metabolism enzymes, not repair factors. They should either be moved to the metabolic section or their indirect relevance to DNA repair (e.g., maintaining dNTP pools) should be explicitly explained.

A: The title of “DNA damage-protection and repair-related genes” has been modified to “DNA damage-protection, repair-related genes and dNTP pools maintenance genes”.

19. CCMEE 029 has two recF orthologs (groups 4008 and 1013), whereas the other strains have only one. This is unusual and potentially interesting , is the extra copy in a genomic island? Is it a recent duplication? Even a brief note in the Discussion would be informative.

A: This has now been addressed in the Discussion.

20. Genes such as recD2 and recF are listed as NA for CCMEE 057 and 064 in the HR section. "NA" could mean genuinely absent, not detected, or not annotated by any tool used. Please clarify , this distinction is biologically critical.

A: This has now been clarified with a legend.

21. The authors note the absence of ggpP/ggpS and gsmT/sdmT genes. This is ecologically interesting because these are osmolytes typical of marine/brackish cyanobacteria, not terrestrial ones. A brief sentence connecting their absence to the desert-adapted lifestyle would strengthen the ecological framing.

A: This concept has now been expanded in the discussion. “which is corroborated by the absence of genes for the production of protective osmolites such as glucosylglycerol, typically found in marine cyanobacteria (Hagemann 2011).”

22. Figure 5 colour scale is dominated by one category. The heatmap colour scale (0–21) is dominated by the HR category (21, 20, 19 genes), which means most other categories appear in nearly the same low-intensity colour, making differences hard to see. Consider either using a log scale or displaying each category row with its own normalised scale.

A: The heatmap color scale has been changed.

23. Figure 6 legend is incomplete. The synteny figure uses several colour-coded modules but the legend does not define all of them clearly. Each colour/module should be explicitly described. Additionally, the statement that the MAA cluster "directly preceded" the scytonemin cluster in CCMEE 057 should include the inter-cluster distance in base pairs, and any evidence for co-regulation (shared promoters, regulatory genes) should be mentioned or its absence noted.

A: Figure 6 caption has now been expanded in relation to the legend and color/modules have been explicitly described. The MAA cluster statement has been updated with “directly preceded the scytonemin cluster by approximately 950 bps”. The co-regulation statement has been added.

24. Typo in CAZyme family name. Supplementary Table 2 uses "GT2_Glyco_tranf_2" (missing an 's'), while the main text uses "GT2_Glyco_transf_2." Please standardise.

A: This has now been fixed.

**Reviews of manuscript FEMSMC-2026-020.R1**

**Reviewer 1: Igor Pessi, University of Helsinki**

**Recommendation: Accept**

Nothing to add.

**Reviewer 2: Amit Srivastava, Purdue University**

**Recommendation: Accept**

The manuscript has improved substantially and is ready for acceptance pending a clean final version.

**Associate Editor Recommendation: Accept**

**EIC Decision: Accept**
